# Effect of Ectomycorrhizal Fungi on the Drought Resistance of *Pinus massoniana* Seedlings

**DOI:** 10.3390/jof9040471

**Published:** 2023-04-14

**Authors:** Min Li, Haoyun Wang, Xizhou Zhao, Wanyan Feng, Guijie Ding, Wenxuan Quan

**Affiliations:** 1 Institute for Forest Resources & Environment of Guizhou, Guizhou University, Guiyang 550025, China; 2 Key Laboratory of Forest Cultivation in Plateau Mountain of Guizhou Province, Guizhou University, Guiyang 550025, China; 3 College of Forestry, Guizhou University, Guiyang 550025, China; 4 Guizhou Provincial Key Laboratory for Information Systems of Mountainous Areas and Protection of Ecological Environment, Guizhou Normal University, Guiyang 550001, China

**Keywords:** *Pinus massoniana*, drought, ectomycorrhizal, soluble sugar, starch

## Abstract

Studies on the dynamics of non-structural carbohydrates (NSCs) play an important role in understanding the mechanisms of plant responses to drought stress. The objective of this study was to assess the influence of ectomycorrhizal fungi (ECMF) on the content and distribution of NSCs in *Pinus massoniana* seedlings under different drought intensities and to further explore the possible mechanism by which ECMF enhances the stress resistance of host plants. We conducted a pot experiment using *P. massoniana* seedlings that were inoculated (M) or non-inoculated (NM) with *Suillus luteus* (*Sl*) under well-watered, moderate, and severe drought stress conditions. The results showed that drought significantly reduced the photosynthetic capacity of *P. massoniana* seedlings and inhibited their growth rate. *P. massoniana* could respond to different degrees of drought stress by increasing the accumulation of NSCs and increasing WUE. However, compared with well-watered treatment, NSCs consumption began to appear in the roots of NM due to the decrease in starch content under severe drought, whereas NSCs content in M seedlings was higher than that in the well-watered treatment, showing that the ability to maintain C balance was higher in M seedlings. Compared with NM, inoculation with *Sl* increased the growth rate and biomass of roots, stems, and leaves under moderate and severe drought. In addition, *Sl* can also improve the gas exchange parameters (net photosynthetic rate, transpiration rate, intercellular CO_2_ concentration and stomatal conductance) of *P. massoniana* seedlings compared with NM seedlings, which was conducive to the hydraulic regulation of seedlings and improved their C fixation capacity. Meanwhile, the content of NSCs in M seedlings was higher. Moreover, the soluble sugar content and SS/St ratio of leaves, roots, and whole plants were higher under drought stress after *Sl* inoculation, indicating that *Sl* could also change the C distribution mode, regulate more soluble sugar to respond to drought stress, which was conducive to improving the osmotic adjustment ability of seedlings, and providing more available C sources for plant growth and defense. Overall, inoculation with *Sl* could enhance the drought resistance of seedlings and promote their growth under drought stress by improving NSCs storage, increasing soluble sugar distribution, and improving the plant water balance of *P. massoniana* seedlings.

## 1. Introduction

The destructive impact of drought on plant growth and yield is global, and temperature and soil aridity are predicted to continue to increase with global climate change [1]. Therefore, we urgently need to find effective ways to improve the drought resistance of plants. The application of ectomycorrhizal fungi (ECMF) plays an important role in improving the growth and drought resistance of host plants. The mechanisms summarized in previous studies include expanding the water and nutrient uptake area and transport efficiency of roots through extraradical mycelium [2], improving stomatal conductance, increasing the photosynthesis efficiency and carbon fixation capacity of host plants [3,4], regulating root development, and changing root architecture by changing auxin metabolism in host plant roots [5]. In addition, it can effectively remove excessive reactive oxygen species (ROS) caused by drought through enhanced antioxidant enzyme activities [6]. However, Colpaert et al. [7] found that in some adverse environments, such as nutritional deficiency, the extent of ECMF development was unfavorable for the growth of *P. sylvestris*. It can be found from the above studies that the response of plants to inoculation with ECMF is highly variable. Therefore, targeted research needs to be carried out on the growth-promoting and stress-resistance mechanisms of ectomycorrhizal fungi on certain fungus–tree combination plants.

As an important afforestation and native timber species in China, *Pinus massoniana* is widely used in afforestation and industrial timber and has ecological value in its wide distribution area. However, due to uneven spatial and temporal distributions, frequent seasonal droughts occur, and the afforestation survival rate and forest productivity of *P. massoniana* decrease due to drought. Therefore, it is necessary to explore effective ways to improve the survival rate and drought resistance of *P. massoniana* seedlings. As a typical mycorrhizal-dependent tree species, *P. massoniana* relies on its symbiotic mycorrhizal fungi to absorb water and nutrients and resist adverse environments. Previous studies have shown that *Suillus luteus* (*Sl*) can promote the growth of *P. massoniana* and improve its drought resistance by promoting nutrient absorption [8], improving root morphology [9], and increasing photosynthetic efficiency and antioxidant enzyme activity [10]. However, there is little consideration or research on factors including the change in non-structural carbohydrate (NSCs) content.

Carbohydrates synthesized through photosynthesis can provide carbon sources for plant growth, among which NSCs mainly include starch (St) and soluble sugar (SS), whose storage and allocation processes have an important impact on plant growth and adaptation to drought stress [11]. At present, there is no unified conclusion on the effect of drought on the NSCs content of different plants. Previous studies have shown that drought stress can reduce NSCs storage by reducing photosynthesis, resulting in plant growth reduction or even death due to carbon starvation [12,13]. At the same time, NSCs depletion also affects the regulation of plant water balance, reducing the ability of plants to transport and retain water [14,15]. However, other studies found that trees can maintain carbon balance through their own strategies, such as reducing growth during drought [16,17]. Jin et al. [18] and Liu et al. [19] found that drought stress had no effect on the NSCs content in plants and even increased its content. Therefore, targeted research is needed to understand how NSCs in tree species respond to drought stress.

ECMF has been shown to affect the change in NSCs content in various tissues of host plants under normal environments and water deficiency, thereby affecting their growth and drought resistance [11,20]. Wang et al. [21] found that inoculation of *Suillus variegatus* could promote the accumulation of NSCs content in each organ of *Pinus tabulaeformis* under drought stress, thus promoting growth and improving drought resistance. However, plant maintenance of ectomycorrhizae also requires the consumption of NSCs. Selosse et al. [22] and Nehls et al. [20] found that the carbon source obtained by ECMF from host plants was up to 20–40% of the carbon assimilated by plants. Compared with species with sparse hyphal development, species with a large amount of hyphal development consume more carbohydrates and have a lower promotion effect on the growth of host plants [7]. In addition, some studies have found that when the carbon resource allocation of symbiotic fungi increases, C metabolism in plant root cells decreases [23,24]. Therefore, more in-depth studies are needed on the effects of ECMF on NSCs accumulation and the water status of host plants, especially under drought-stress conditions. It would be helpful to further understand the mechanism by which ECMF enhances the drought resistance of host plants.

To understand whether *Sl* can affect the growth and drought resistance of *P. massoniana* by regulating the change in NSCs content and distribution, we studied the growth, gas exchange parameters, and contents of soluble sugar and starch in plant tissues of *P. massoniana* seedlings inoculated or uninoculated with *Sl* under different drought levels. The results will help to further explore the potential mechanism of *Sl* in promoting the growth and drought resistance of *P. massoniana* seedlings and provide a theoretical basis for enhancing the afforestation survival rate and forest productivity.

## 2. Materials and Methods

### 2.1. Plant Material and Growth Conditions

The test site is a greenhouse at Guizhou University (26°26′ N, 106°39′ E) in Guiyang, Guizhou Province, China. *P. massoniana* seeds were provided by the Duyun *Pinus massoniana* seed orchard, sterilized with 0.05% KMnO_4_ for 30 min, washed, and spread into germinating boxes with presterilized vermiculite, germinated at 25 °C for 28 days, and then transplanted into plastic pots (diameter and depth were 20 cm and 16 cm, respectively) filled with 4 kg of growth medium. Each pot was planted with 3 seedlings. The growth medium consisted of soil, perlite, and vermiculite (4:1:1, v/v/v, pH 4.65), which was sterilized at 121 °C for two hours before transplantation. The main nutrient characteristics of the growth medium were 44.49 g·kg^−1^ organic matter, 56.34 mg·kg^−1^ available P, 157.5 mg·kg^−1^ available N, and 35.85 mg·kg^−1^ available K.

### 2.2. Fungal Preparation and Inoculation

*Suillus luteus* (*Sl*) was isolated from the sporophore, which was collected from the undergrowth of a *Pinus massoniana* forest in Guizhou Province. The strain was first cultivated on Pachlewski (PACH) solid medium for two weeks. Then, 4 blocks of media (6 mm in diameter) were inoculated in 300 mL of PACH liquid medium. After 4 weeks of culture shaking (dark condition, 80 r·min^−1^, 25 °C), the mycelium was homogenized using a homogenizer.

**Figure 1:**
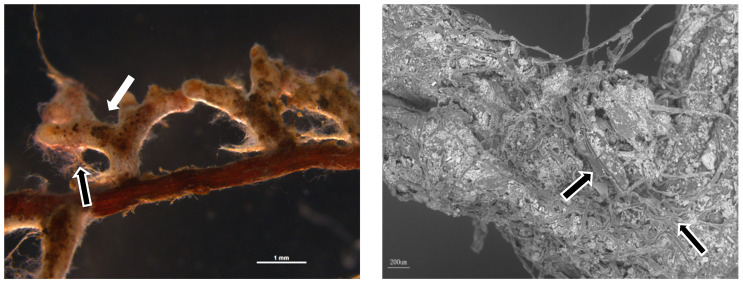
Colonization of *Suillus luteus* (*Sl*) on the root of *Pinus massoniana*. Dichotomous branches (white arrow), extraradical mycelium (black arrow).

Seedlings were transplanted, and *Sl* was inoculated at the same time (Figure 1). Fifty milliliters of inoculum was poured into the rhizosphere of each seedling, and the same amount of blank culture solution was applied to the uninoculated seedlings. Then, the seedlings were cultured in the greenhouse and watered every two days (the average temperature and relative humidity of the greenhouse were 25 °C and 80%, respectively).

### 2.3. Experimental Design

One-year inoculated seedlings (M) and non-inoculated seedlings (NM) with basically the same growth were assigned to three different drought treatments, and the three drought treatments were well-watered/control (T1), moderate drought (T2), and severe drought (T3), with 80%, 50%, and 35% of the field capacity, respectively, and 24 inoculated and uninoculated seedlings in each drought gradient. 

The weighing method was used to maintain the water content required by the test at 6 p.m every day. After 60 days, the test was finished, and samples were taken for determination.

### 2.4. Index Determination

#### 2.4.1. Gas Exchange

Leaf instantaneous gas exchange parameters, including the net photosynthetic rate (Pn), transpiration rate (Tr), intercellular CO_2_ concentration (Ci), stomatal conductance (Gs), and leaf water use efficiency (WUE=Pn/Gs), were measured before sampling. The method was described by Li et al. [25].

#### 2.4.2. Seedling Growth

Seedling ground diameter and height were measured on the first and 60th days of water treatment to calculate the net increase in seedling growth during the water treatment.

#### 2.4.3. Colonization, Biomass, Root Vitality, and NSCs Content

The harvested whole seedlings were carefully washed with running water, dried with absorbent paper, and then used to determine ECM fungal colonization, root vitality, biomass, soluble sugar (SS), and starch (St).

Determination of biomass, the content of SS and St: Whole seedlings were quickly separated into three parts: roots, stems, and leaves. The seedlings were dried at 80 ℃ to constant weight (DW), and then the biomass of each tissue was weighed. After that, the tissues were crushed and screened (0.42 mm) to determine the SS and St contents in each tissue, and the NSCs content for each tissue was the sum of the SS and St contents. The ratio of soluble sugar to starch (SS/St) was calculated to explain the NSCs conversion dynamics. Whole plant total non-structural carbohydrate content (TNC) was the sum of the NSCs contents of each tissue, as well as whole plant soluble sugar (TSS) and whole plant starch (TSt). The method used was described by Hansen and Møller [26], with a few modifications.

Determination of relative water content (RWC): a small amount of mature leaves was weighed as fresh weight (FW), and turgid weight (TW) was obtained after leaves were soaked in distilled water to constant weight, and then dried at 80 °C to constant weight to obtain dry weight (DW), and the RWC of leaves was calculated.

RWC = [(FW − DW)/(TW − DW)] × 100%

Determination of ECM fungal colonization: The grid line intersection method was used to count the colonization, as described by Wang et al. [21].

Determination of root vitality: The triphenyltetrazolium chloride (TTC) reduction method was used to determine root vitality [27].

### 2.5. Statistical Analysis

All data were tested for homogeneity before two-way ANOVA (testing the effects of ECMF (*Suillus luteus*) and drought level on growth, gas exchange parameters, NSCs contents in each tissue and whole plant, root vitality, and RWC of M and NM seedlings, *p* < 0.05, 0.01) and one-way ANOVA (Tukey’s HSD test was used to detect significant differences in each parameter, *p* < 0.05) with SPSS 20.0 software (SPSS Inc. Chicago, IL, USA). Origin Pro8.5 (OriginLab Corporation, Northampton, MA, USA) was used to create figures. The correlation between growth and physiological-biochemical indexes was evaluated using Pearson’s correlation coefficient at *p* < 0.05 with R3.6.3 software.

## 3. Results

### 3.1. The Growth of P. massoniana

**Figure 2:**
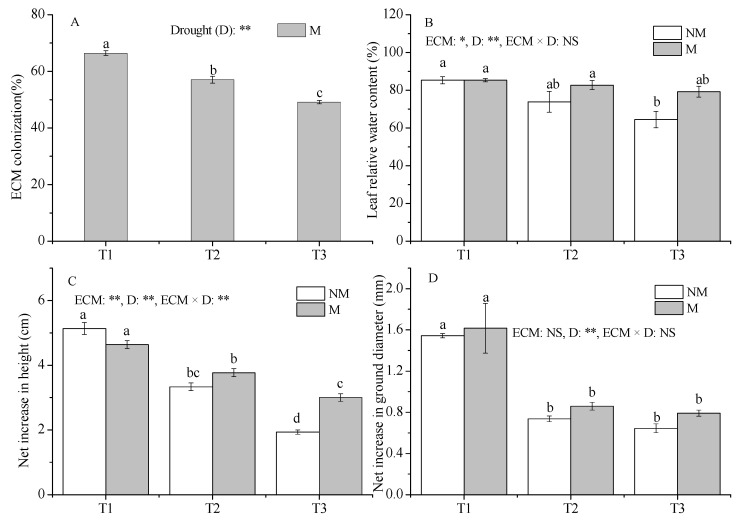
Effect of ECMF and drought on the growth, colonization rate, and water status of *P. massoniana*. Values (means ± SEs) followed by different letters among treatments indicate significant differences at the 5% level, and the same letter indicates no significant difference. NS, not significant, * *p* < 0.05; ** *p* < 0.01. T1, well-watered, T2, moderate drought, and T3, severe drought. NM, no *Sl* inoculation, M, *Sl* inoculation. D, drought treatment. The letters denote (**A**) ECMF colonization; (**B**) leaf relative water content; (**C**) net increase in seedlings height; (**D**) net increase in seedlings ground diameter.

The roots of all inoculated seedlings were colonized by *Sl*, and ECMF colonization decreased significantly with increasing drought intensity (Figure 2A).

Drought reduced the RWC of both NM and M seedlings, and the RWC of NM seedlings in the T3 treatment decreased significantly compared with that in T1 treatment (Figure 2B). Compared to NM, *Sl* increased RWC to some extent under the same drought treatments. 

The net increase of the ground diameter and height of seedlings decreased significantly as the drought intensity increased (*p* < 0.05) (Figure 2C,D). Compared with NM seedlings, inoculation with *Sl* improved the net increase of height and diameter in all treatments and significantly increased the net increase of height under the T3 treatment (*p* < 0.05).

### 3.2. Biomass of P. massoniana

**Figure 3:**
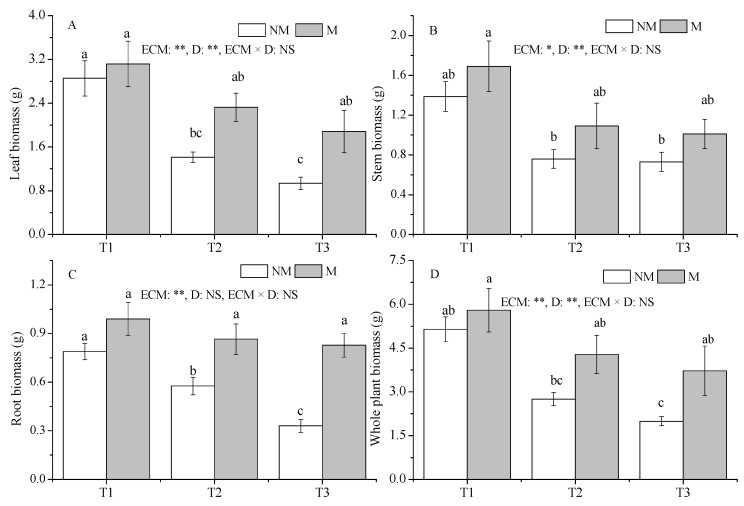
Effect of ECMF and drought on the biomass of *P. massoniana.* Values (means ± SEs) followed by different letters among treatments indicate significant differences at the 5% level, and the same letter indicates no significant difference. NS, not significant, * *p* < 0.05; ** *p* < 0.01. T1, well-watered, T2, moderate drought, and T3, severe drought. NM, no *Sl* inoculation, M, *Sl* inoculation. D, drought treatment. The letters denote (**A**) leaf biomass; (**B**) stem biomass; (**C**) root biomass; (**D**) whole plant biomass.

Compared with the T1 treatment, the T3 treatment significantly reduced the biomass of roots, stems, and leaves of the NM seedlings (*p* < 0.05), as well as the whole-plant biomass of the NM seedlings (Figure 3A–D). Drought had no significant effect on various tissues and whole plant biomass of M seedlings. Compared with NM seedlings, inoculation with *Sl* significantly improved leaf biomass and total plant biomass under the T3 treatment and significantly improved root biomass under the T2 and T3 treatments (*p* < 0.05).

### 3.3. Gas Exchange Parameters of P. massoniana

The Pn, Gs, Ci, and Tr of leaves decreased significantly as the drought gradient increased (*p* < 0.05) (Table 1). The inoculation of *Sl* significantly increased Ci and Tr under the T2 and T3 treatments by 28.88% and 21.01% (Ci), 40.88% and 50.22% (Tr), respectively, compared with NM seedlings (*p* < 0.05). Similarly, *Sl* inoculation significantly increased Pn under the T3 treatment. However, inoculation with *Sl* had no significant effect on Gs in all treatments but still had a promoting effect on Gs. In contrast, WUE increased significantly with increasing drought intensity, and inoculation with *Sl* significantly decreased WUE in the T2 and T3 treatments compared with NM seedlings (*p* < 0.05).

### 3.4. The Non-Structural Carbohydrates

**Figure 4:**
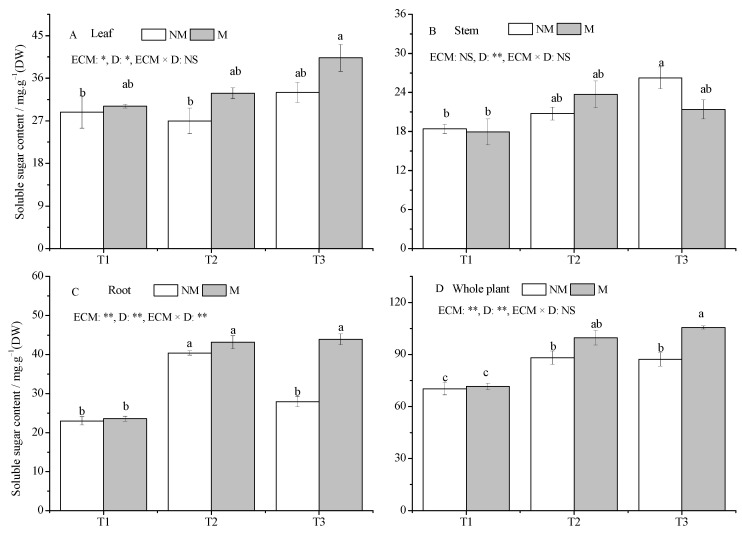
Effect of ECMF and drought on soluble sugar contents of various tissues and whole plants of *P. massoniana.* Values (means ± SEs) followed by different letters among treatments indicate significant differences at the 5% level, and the same letter indicates no significant difference. NS, not significant, * *p* < 0.05; ** *p* < 0.01. T1, well-watered, T2, moderate drought, and T3, severe drought. NM, no *Sl* inoculation, M, *Sl* inoculation. D, drought treatment. The letters denote (**A**) leaf soluble sugar; (**B**) stem soluble sugar; (**C**) root soluble sugar; (**D**) whole plant soluble sugar.

Drought increased the SS content in all seedling tissues, except for a slight decrease in NM seedlings under the T2 treatment compared with the T1 treatment (Figure 4A–C). Among them, the SS contents of stems in the T3 treatment and roots in the T2 treatment were significantly higher than those in the T1 treatment in NM seedlings. Similarly, the M seedlings roots and leaves in the T3 treatment had higher SS content compared with the T1 treatment (*p* < 0.05). The inoculation of *Sl* significantly increased the root SS content compared with that of NM seedlings under the T3 treatment (*p* < 0.05).

The SS content of whole plants was significantly increased under drought stress, NM seedlings in the T2 and T3 treatments were increased by 25.48% and 24.12% compared with the T1 treatment, and M seedlings increased by 39.20% and 47.50% (Figure 4D). Compared with NM seedlings, the inoculation of *Sl* significantly increased the whole plant SS content by 21.10% under the T3 treatment (*p* < 0.05).

**Figure 5:**
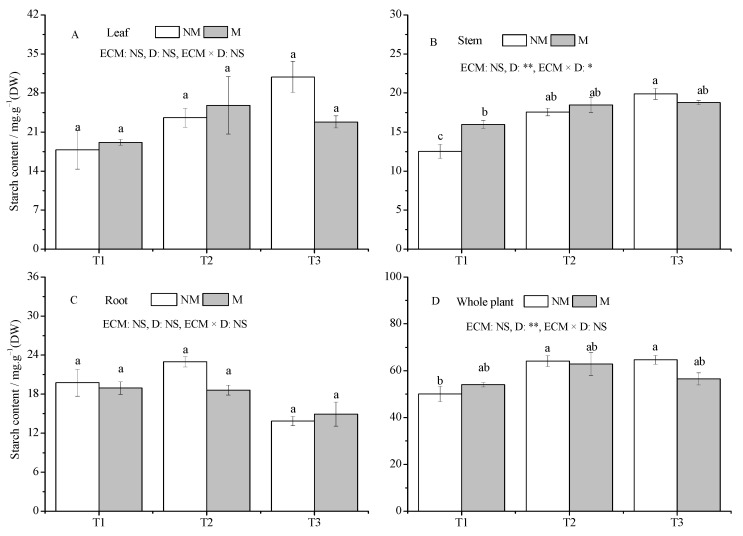
Effect of ECMF and drought on the starch contents of various tissues and whole plants of *P. massoniana.* Values (means ± SEs) followed by different letters among treatments indicate significant differences at the 5% level, and the same letter indicates no significant difference. NS, not significant, * *p* < 0.05; ** *p* < 0.01. T1, well-watered, T2, moderate drought, and T3, severe drought. NM, no *Sl* inoculation, M, *Sl* inoculation. D, drought treatment. The letters denote (**A**) leaf starch; (**B**) stem starch; (**C**) root starch; (**D**) whole plant starch.

Compared with the T1 treatment, the starch content of stems and whole plants increased significantly in NM seedlings under drought conditions (*p* < 0.05), but remained relatively stable in all tissues and whole plants of M seedlings in all treatments (Figure 5A–D). Inoculation with *Sl* had no significant effect on starch content in all treatments.

**Figure 6:**
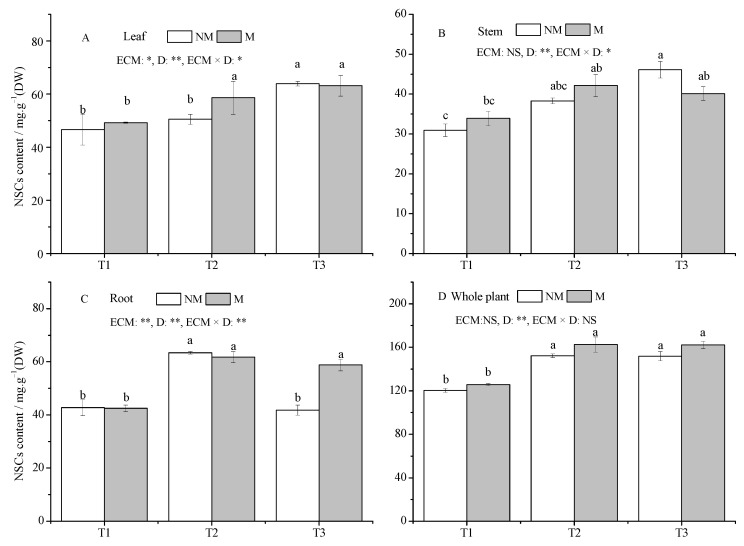
Effect of ECMF and drought on the NSCs contents of various tissues and whole plants of *P. massoniana.* Values (means ± SEs) followed by different letters among treatments indicate significant differences at the 5% level, and the same letter indicates no significant difference. NS, not significant, * *p* < 0.05; ** *p* < 0.01. T1, well-watered, T2, moderate drought, and T3, severe drought. NM, no *Sl* inoculation, M, *Sl* inoculation. D, drought treatment. The letters denote (**A**) leaf NSCs (non-structural carbohydrates); (**B**) stem NSCs; (**C**) root NSCs; (**D**) whole plant NSCs.

Drought increased the NSCs content of all tissues and whole plants, except that NM seedlings roots in the T3 treatment had lower NSCs content compared with the T1 treatment (Figure 6A–D). A significant increase in whole plant NSCs content was detected under drought stress. NM seedlings in the T2 and T3 treatments increased by 26.50% and 26.17%, respectively, and M seedlings increased by 29.37% and 29.03%, respectively, compared with the T1 treatment (*p* < 0.05). Compared with NM seedlings, *Sl* inoculation significantly increased the root NSCs content by 40.65% under the T3 treatment, and increased the leaf NSCs content by 15.93% under the T2 treatment (*p* < 0.05). In addition, the inoculation of *Sl* had no significant effect on other tissues and whole-plant NSCs contents under all treatments.

**Figure 7:**
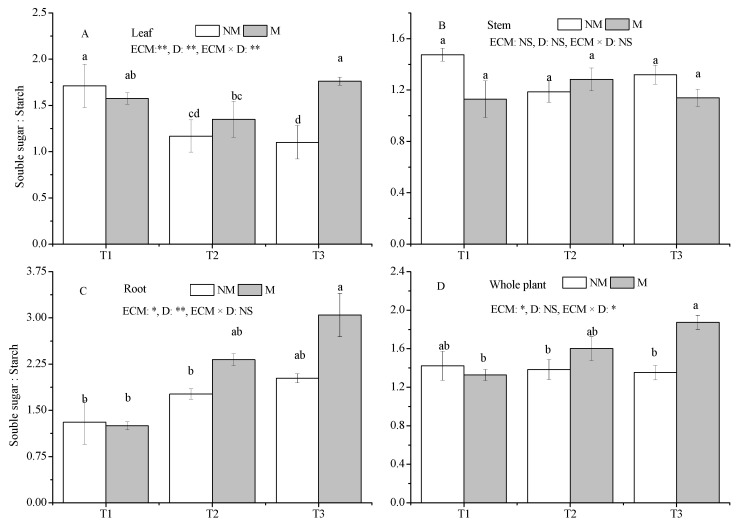
Effect of ECMF and drought on the SS/St ratio of various tissues and whole plants of *P. massoniana.* Values (means ± SEs) followed by different letters among treatments indicate significant differences at the 5% level, and the same letter indicates no significant difference. NS, not significant, * *p* < 0.05; ** *p* < 0.01. T1, well-watered, T2, moderate drought, and T3, severe drought. NM, no *Sl* inoculation, M, *Sl* inoculation. D, drought treatment. The letters denote (**A**) ratio of soluble sugar to starch of leaf; (**B**) ratio of soluble sugar to starch of stem; (**C**) ratio of soluble sugar to starch of root; (**D**) ratio of soluble sugar to starch of whole plant.

Compared with the T1 treatment, the T2 and T3 treatments significantly decreased the SS/St ratio of leaves in NM seedlings, whereas the T3 treatment significantly increased the SS/St ratio of roots and whole plants in M seedlings (*p* < 0.05) (Figure 7A,C,D). In addition, no significant effect of water deficiency on the SS/St ratio of other tissues and whole plants of both NM and M seedlings was found (Figure 7A–D). It is likely that *Sl* had no effect on the SS/St ratio in all treatments, except a significant increase in leaf and whole-plant SS/St ratio was detected in the T3 treatment compared with NM seedlings (*p* < 0.05) (Figure 7A,D).

### 3.5. Root Vitality

**Figure 8:**
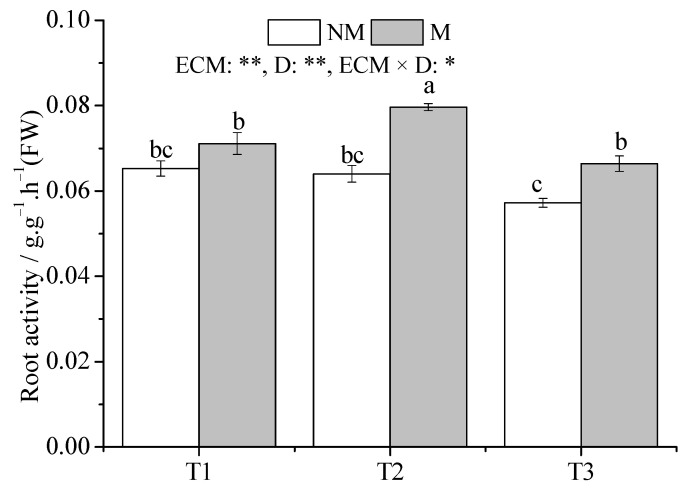
Effect of ECMF and drought on root vitality. Values (means ± SEs) followed by different letters among treatments indicate significant differences at the 5% level, and the same letter indicates no significant difference. NS, not significant, * *p* < 0.05; ** *p* < 0.01. T1, well-watered, T2, moderate drought, and T3, severe drought. NM, no *Sl* inoculation, M, *Sl* inoculation. D, drought treatment.

The root vitality of M seedlings in the T2 treatment was higher than that in the T1 treatment (*p* < 0.05) (Figure 8). In addition, drought had no significant effect on seedlings root vitality. Inoculation with *Sl* significantly increased root vitality compared with that of NM seedlings under the T2 and T3 treatments (*p* < 0.05).

### 3.6. Correlation Analysis

**Figure 9:**
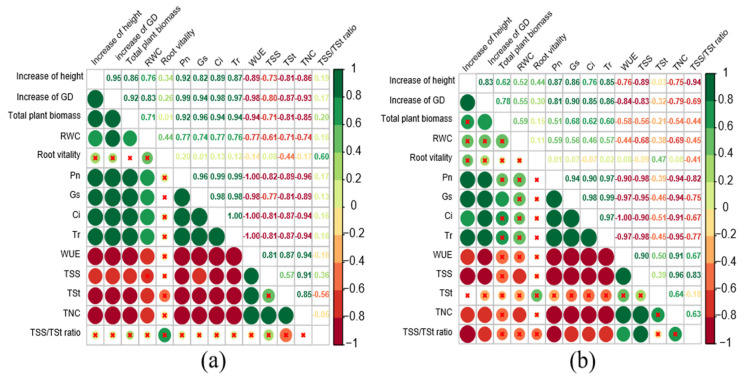
Pearson correlation coefficients among growth, total biomass, water status, and other physiological and biochemical indexes in NM seedlings (**a**) and M seedlings (**b**) × represents *p* > 0.05, with no significant correlation. TSS/TSt ratio, the ratio of whole plant soluble sugar to whole plant starch; GD, ground diameter.

Pearson correlation analysis was performed for seedlings growth, total biomass, RWC, and other physiological and biochemical indexes (Figure 9a,b). The results showed that inoculation with *Sl* changed the correlation between each index and growth, biomass, and RWC. Among them, the growth, total biomass, and RWC of NM seedlings were significantly positively or negatively correlated with other indexes except for root activity and the ratio of TSS/TSt (*p* < 0.05). The net increase of height and ground diameter of M seedlings were significantly positively correlated with gas exchange parameters and negatively correlated with WUE, TSS and TNC; RWC was only negatively correlated with TSS and TNC, and whole-plant biomass was only positively correlated with Gs (*p* < 0.05).

## 4. Discussion

### 4.1. Inoculation Can Improve the Hydraulic Regulation of Seedlings and Maintain More Water

Stomatal closure is considered to be one of the first effects of drought on plants, which helps to prevent or slow the water loss of tissues [28]. Similarly, we found that *P. massoniana* can respond to different degrees of drought by improving WUE and reducing water loss by reducing Gs and Tr, whereas *Sl* can improve the gas exchange capacity under drought, indicating that ECMF can perform hydraulic regulation under stress conditions. This may be because larger root systems can absorb more water, or the extension hyphae expand the water and nutrient absorption area [29,30], and the higher RWC in the inoculated seedlings can confirm this. In addition, the increase in stomatal conductance also helps to increase the absorption of CO_2_, thus increasing the net photosynthetic rate. Therefore, although drought limits the stomatal conductance of *P. massoniana*, which will reduce the ability of C acquisition and inhibit plant growth [31], the inoculated seedlings can still synthesize more carbohydrates needed for respiration than the uninoculated seedlings under the same degree of stress, maintaining basic metabolism and defense ability. So as to promote the growth of seedlings under stress, which showed that the inoculated seedlings had higher net increase of height and ground diameter, and biomass. Symbiotic relationships with ECMF have also been shown to promote host plant growth in other studies [32,33]. Overall, the application of *Sl* may be beneficial to increase plant water balance and improve the photosynthesis of *P. massoniana* seedlings, thereby enhancing their carbon acquisition ability and drought resistance.

### 4.2. Inoculation Altered the Content and Distribution of NSCs in Seedlings under Drought Stress

Studies have shown that drought has a significant effect on plant NSCs content, which can restrict plant photosynthesis through the closure of plant stomata, resulting in the reduction of photosynthetic products. After a long period of drought, plants begin to mobilize and utilize the stored soluble carbohydrates in plants to maintain plant growth [34]. However, an increase in NSCs has been observed in some cases because drought has a stronger effect on growth and respiration than on photosynthesis [35,36]. This may be the reason for the increase in NSCs content in various tissues and whole plants of *P. massoniana* seedlings (except NSCs content in roots of NM seedlings under severe stress). NSCs play an important role in energy metabolism, transport, and osmoregulation in plants [15]. The way that plants adapt to a stressful environment by increasing the storage of NSCs is beneficial to reduce damage and promote rapid repair [37,38], which may also be an effective way for *P. massoniana* seedlings to resist drought stress.

In addition, the influence of severe drought on plant NSCs is dependent on its duration. A short drought decreased the starch concentration in the photosynthetic organs of *Chamaecyparis obtusa* and increased the soluble sugar concentration. However, after 3 months of long-term severe drought, the soluble sugar and starch contents in all tissues of seedlings were lower than those of the watering treatment [39]. Therefore, we speculated that the duration of the drought was not long enough for *P. massoniana*, which had relatively strong drought tolerance, and the NSCs content in seedlings did not show obvious consumption. The results showed that *P. massoniana* seedlings could maintain enough photosynthesis to exceed the compensation point under prolonged water deprivation conditions.

The increase in NSCs storage is an effective ecological strategy for plants to adapt to adversity, and the change in NSCs content greatly affects the ability of plants to cope with and recover from adversity [40]. Compared with leaves, stems and roots are more likely to suffer carbon starvation due to the depletion of reserves [41]. In this study, the content of NSCs in the roots of NM seedlings under severe drought showed a decreasing trend compared with the well-watered treatment, which was mainly caused by the decrease in starch content. We speculated that the root system of NM would have carbon imbalance under long-term severe drought, with the risk of carbon hunger, and could not adapt to severe drought. Sala et al. [42] believed that one of the causes of carbon starvation in plant local organs was the obstruction of NSCs transmission, which was consistent with our results. NSCs in NM seedlings accumulated more in leaves and stems, leading to a depletion of starch in roots, even though the increase in soluble sugar did not buffer this change in starch under severe drought. This may be due to the significant decrease in gas exchange parameters and water conductivity under drought conditions, which is not conducive to the transport of mobile carbon and maintenance of carbon reserves [43]. However, under severe drought conditions, the content of NSCs in the tissues of seedlings inoculated with *Sl* was continuously higher than that of the well-watered treatment, which may be because ECMF alleviated the restriction of drought stress on plant water balance and photosynthesis, thus improving the ability to maintain carbon balance in all organs of *Pinus massoniana* seedlings to improve the ability to cope with drought.

In addition, there was no significant difference in NSCs content in the roots, stems, leaves, and whole seedlings of NM and M seedlings under the same drought treatment, except that the NSCs content in the stem and roots of M seedlings was significantly higher than that of NM seedlings under moderate and severe drought. This indicated that *Sl* inoculation could effectively accelerate the accumulation of NSCs in roots under severe drought, especially the accumulation of soluble sugar, to promote root and *Sl* growth and water absorption under stress conditions, which could be proven by higher root vitality and biomass in M seedlings. The reason for this phenomenon may be that mycelial and plant root growth are strong sinks of carbohydrates, and carbohydrate transport from leaves to roots is closely related to carbon assimilation and moisture state [44,45]. Compared with NM, the higher stomatal conductance in M seedlings increased the absorption and fixation of CO_2_, as well as the absorption and transport of water, which was beneficial to transfer more NSCs to roots to ensure root growth and absorption capacity under drought conditions, and could also provide carbohydrates for symbiotic ECMF. In addition, except for the soluble sugar content and the ratio of SS/St in the stem of M seedlings being slightly lower than that of NM under severe stress, the soluble sugar content and the ratio of SS/St in all tissues and whole seedlings of M were higher than that of NM under water deficiency conditions, showing that *Sl* could change the composition of NSCs and regulate more soluble sugar in response to drought stress. Correlation analysis and SS/St ratio results also showed that NM mainly increased NSCs content by accumulating starch in response to drought stress, whereas M seedlings mainly increased NSCs content by accumulating soluble sugar. Soluble sugar can not only be used as a C source for plants, but also participates in osmotic regulation as an osmotic regulatory substance to maintain plant cell turgor; it also promotes the transfer of carbohydrates in the plant [46,47], thus improving the drought resistance of M seedlings. Similarly, Wang et al. [21] also found that ECMF could promote the growth of *P. tabulaeformis* under water deficiency by increasing the soluble sugar content and the ratio of soluble sugar to starch. At the same time, the SS/St ratio in the leaves and stems of NM seedlings decreased under drought stress compared with the well-watered treatment, whereas the SS/St ratio in M seedlings increased under severe stress, which suggested that NSCs reserves in the leaves and stems of NM seedlings were mainly used for storage under drought stress. However, M seedlings utilize NSCs reserves, which are beneficial for meeting carbon requirements for the growth and production of defensive compounds [48]. In general, the application of *Sl* may change the storage and distribution patterns of host plant NSCs to make them more effective in response to drought stress. Of course, *P. massoniana* can form symbiotic relationships with more than one species of fungus in the field. Symbiotic fungi absorb water and nutrients for the host plant and, in return, obtain carbohydrates from the host [49]. Drought stress may lead to C restriction, and plants may face carbon allocation tradeoffs between maintaining water balance through NSCs storage and sustaining symbionts [15]. At this time, the effects of a large number of symbiotic fungi on host drought resistance, especially whether and under what circumstances ECMF carbon demand affects plant NSCs storage and drought resistance, are unknown, but deserve attention. This also provides interesting ideas for later research.

## 5. Conclusions

In this study, drought inhibited the growth and photosynthetic capacity of both NM and M seedlings, and seedlings responded to drought stress by increasing the NSCs content. *Sl* could improve the drought resistance and growth of *Pinus massoniana* seedlings by hydraulic regulation, promoting the accumulation of NSCs, maintaining the C balance in seedlings tissues, and changing the distribution pattern of NSCs (regulating more soluble sugars) compared to NM seedlings. These findings further demonstrate the importance of NSCs in plant drought resistance and help us further understand the relationship between ECMF and host plant drought resistance. The exact mechanism by which ECMF promotes growth and stress resistance will be analyzed in combination with molecular biology methods in our further studies, exploring the potential of ECMF in improving plant stress resistance and promoting sustainable forest management.

## Figures and Tables

**Table 1:** Effect of ECMF and drought on the photosynthesis of *P. massoniana* seedlings.

Drought Intensity	ECM Treatment	Pn (umol CO_2_ m^−2^ s^−1^)	Ci (umol CO_2_ mol^−1^)	Gs (mol H_2_O m^−2^ s^−1^)	Tr (mmol H_2_O m^−2^ s^−1^)	WUE
T1	NM	14.09 ± 0.08 a	290.99 ± 5.24 a	0.42 ± 0.04 a	3.93 ± 0.12 a	36.62 ± 3.13 d
	M	14.38 ± 0.34 a	295.52 ± 2.91 a	0.44 ± 0.01 a	4.12 ± 0.04 a	32.98 ± 1.51 d
T2	NM	4.63 ± 0.18 bc	160.08 ± 3.41 d	0.04 ± 0.00 b	0.99 ± 0.05 c	125.08 ± 2.27 a
	M	5.59 ± 0.40 b	206.31 ± 2.63 b	0.05 ± 0.00 b	1.41 ± 0.10 b	107.69 ± 1.73 b
T3	NM	3.79 ± 0.02 d	152.75 ± 1.19 d	0.03 ± 0.00 b	0.94 ± 0.00 c	129.35 ± 0.77 a
	M	4.32 ± 0.08 c	184.84 ± 2.28 c	0.05 ± 0.00 b	1.40 ± 0.01 b	91.87 ± 2.32 c
	ECM	*	**	NS	**	**
	D	**	**	**	**	**
	ECM × D	NS	**	NS	NS	**

Values (means ± SEs) followed by different letters among treatments indicate significant differences at the 5% level; NS, not significant * *p* < 0.05; ** *p* < 0.01. D means drought treatment. Pn, net photosynthetic rate; Tr, transpiration rate; Ci, intercellular CO_2_ concentration; Gs, stomatal conductance; WUE, leaf water use efficiency.

## Data Availability

The original data presented in this study are included in the article. Further inquiries can be directed to the corresponding author.
